# Impact of management’s irrational expectations on corporate tax avoidance: A mediating effect based on level of risk-taking

**DOI:** 10.3389/fpsyg.2022.993045

**Published:** 2022-11-18

**Authors:** Lingyu Li, Qing Wu

**Affiliations:** School of Management, Shanghai University of Engineering Science, Shanghai, China

**Keywords:** management, irrational expectations, risk-taking, tax avoidance, tone

## Abstract

Frequent tax avoidance incidents have caused huge losses to corporate reputation and corporate value. Research is required on whether and how the irrational judgment of management, a powerful factor in corporate decision-making, affects corporate tax avoidance behavior. Taking all A-share listed companies from 2006 to 2020 as a sample, this paper empirically tests the relationships among management’s irrational expectations, level of corporate risk-taking, and level of corporate tax avoidance using an fixed effects regression model (FEM). The results of the three-stage regression model and Sobel test suggest that the level of corporate risk-taking plays a mediating role between managers’ irrational expectations and Corporate tax avoidance. The managers’ stockholding plays a moderating role in this process. This study also finds evidence that the irrational expectations of management lead to an increase in levels of research and development manipulation, which indirectly increases the level of corporate tax avoidance. Therefore, to control the risk caused by managers’ risky decisions, such as R&D manipulation and tax avoidance, it is necessary to lessen the effects of irrational expectations of management, and the management equity incentive plan has been identified as a reliable method in the risk reduction process.

## Introduction

In recent years, research on corporate governance has paid more and more attention to the personal characteristics of managers (Hribar et al., 2017). Much of this research has drawn on the development of behavioral finance theory in accordance with the hypothesis of the “rational economic man” in traditional economics. Tax avoidance decisions, an enterprise behavior with complex characteristics that brings uncertainty to the future development of an enterprise, are easily influenced by the subjective consciousness of enterprise managers during the process of implementation. The upper echelons theory in behavioral finance emphasizes that the internal and external environment of enterprises is complex. Even within a limited range of matters, managers’ resources are finite, and the degree of attention will vary from person to person. The cognitive structure and values developed by managers through their early learning and work experience have a decisive impact on how they interpret and process relevant information; personal characteristics; and personal abilities have a profound effect on the strategic choices of enterprise managers, which are further manifested through the business decisions of their enterprises (Tsai et al., 2022). When there is a discrepancy between management perceptions and other expectations of an enterprise’s prospects, the management team’s ability to operate will be affected, as will their self-perception and business choices in relation to the enterprise’s further development. This impact is likely to lead to risk-taking, such as the decision to avoid tax. Following such a decision, the discrepancy in management judgment is likely to continue to interfere with decision-making.

Tax is a compulsory payment made by natural persons or enterprises to the government. Under the constraints of appropriate laws and regulations, it has become one of the most important sources of revenue for all countries, and is of great significance to the stable and orderly development of their economies China’s corporate income tax revenues rose from 21.72% in 2015 to 24.3% in 2021, making it the country’s second-largest source of tax revenue. This steady rise in enterprise income tax revenue has had a positive impact on the government’s reduction of the fiscal deficit and optimization of resource allocation. However, for tax-paying enterprises seeking to maximize their own interests, it is inevitable that tax will be regarded as a burden that reduces net income; this perception is also found in developed countries, not least the United Kingdom and the United States. In the developing countries of Southeast Asia, which are seeking innovation, transformation, and modernization, tax avoidance, and derivative problems are increasingly serious. Most enterprises engage in tax planning by using loopholes in tax laws and regulations. Such planning complicates simple transactions and hides their essence in order to reduce the company’s tax burden. In 2020, eBay multinational companies paid only GBP 1.6 million in taxes on earnings of GBP 2 billion. In 2021, according to Sina Finance news, Huali Group, the “registered arbitrage God” of A-share companies listed in China, transferred RMB 3.7 billion of its profits to avoid taxes. According to the US Treasury Department, the tax avoidance of large companies, including influential companies, such as Bank of America, Citigroup, and Morgan Stanley, costs the country nearly USD 100 billion a year. In China, according to the statistics issued by the tax authorities, tax avoidance losses caused by transfer pricing, multinational transactions, and other transactions are as high as 30 billion yuan per year.

Because of the huge impact of tax avoidance, many studies have focused on corporate tax avoidance, notably from the perspectives of political connections, governance structure, investor monitoring, and macroeconomic factors (Khurana and Moser, 2013; Evangelos et al., 2017; Chen et al., 2021). Few studies have focused on the impact of management expectations, a subjective element, on tax avoidance activities. Although a few researchers, in China and internationally, have discussed tax avoidance from this perspective such as the formal sector’s perception of market competition (Damayanti et al., 2021), their discussions have remained theoretical because of the difficulty of observing management expectations and the lack of transparency of enterprise information. With the continuous improvement of relevant norms such as the Rules for the Content and Format of Information Disclosure of Companies Publicly Issuing Securities and the continuous progress of text information analysis software and statistical technology, capital market participants can now more accurately understand and explain the emotional expression of management (Miao et al., 2020) to the extent of quantifying the irrational bias of management decision-making in the operation of listed companies (Iwanty and Surjandari, 2022). The present study takes this opportunity to explore the impact and mechanism of management’s irrational expectations on levels of corporate tax avoidance, analyzing how they affect corporate tax avoidance activities through different transmission mechanisms. The findings enrich the academic literature on the influencing factors of corporate tax avoidance behavior.

Shanghai and Shenzhen A-share listed companies are a representative part of China’s economy. Their business data are easily accessible and the disclosure content is abundant. Drawing on a sample of companies listed on the Shanghai and Shenzhen Stock Exchanges from 2006 to 2020, this study tests empirically how management’s irrational expectations affect corporate tax avoidance. The results show (1) that the greater the management’s irrational expectations, the greater the tendency of an enterprise to take higher risks, which helps to stimulate corporate tax avoidance behavior; and (2) that the managers’ stockholding plays a moderating role in the relationship between the irrational expectations of management and tax avoidance behavior, the impact of irrational expectations being more significant for enterprises with less managers’ stockholding ratio. Further analysis clarifies the impact of management’s irrational expectations on corporate research and development (R&D) manipulation. The results indicate that managers’ irrational expectations can result in manipulation of transactions, which can stimulate corporate tax avoidance.

This paper mainly adopts the empirical research method. It can be divided into five steps. Step 1—Establish the scientific hypothesis; Step 2—Choose the proper variables; Step 3—Select the optimal data for the analysis etc.; Step4—Establish fixed effect regression model and analyze results; and Step 5—Discuss and remark this research.

## Establish the scientific hypothesis

An objective and rational view of a company’s aggressive tax avoidance behavior cannot be separated from a cost–benefit analysis of that behavior. Although tax avoidance behavior can protect a company’s cash flow and income in the short term, for tax avoidance purposes the company must go through a complex process of tax planning, which incurs costs that are both direct and indirect. Direct costs include explicit costs, such as tax planning, external agency consultation, and transaction complexity fees. Indirect costs, which are difficult to quantify in the short term but which affect the long-term survival and development of the company, include an increase in principal–agent problems, damage to corporate reputation, and tax inspection risks.

The common phenomenon of the separation of ownership and management makes it possible for management to cover up opportunism with secret tax avoidance activities, which leads to the risk of management pursuing private interests (Badertscher et al., 2013) of limited compensation, the management of listed companies may be insufficiently motivated to form a common interest with shareholders, which makes it difficult to resolve agency conflict and creates objective conditions for the management to use their own power to carry out tax planning and tax avoidance activities. At the same time, the personal behavioral tendencies of management, together with the management style forged by their own growth and practical experience (Brunsson, 1982; Bertrand and Schoar, 2003), can generate a decision-making bias that is the subjective motivation of tax avoidance behavior (Park and Kim, 2020). Scholars in the field of corporate finance have noted that managers play a key role in corporate tax avoidance decisions. Even if managers do not directly make the decision to avoid tax, their power and influence are enough to form the corresponding management tone and to affect tax avoidance behavior (Dyreng et al., 2010). As with the planning of the work priorities of various functional departments of the enterprise, the formulation of enterprise resource allocation strategies, and the planning of incentive standards and methods for financial and tax managers, these powers provide opportunities for management to act on personal preferences for tax avoidance activities (Christensen et al., 2015). These considerations show that there is a close relationship between managers and corporate tax avoidance (Zhang et al., 2020).

Given the relationship between the personal characteristics of managers and corporate tax avoidance, scholars have called for detailed study of whether and how the subjective behavioral tendencies and psychological bias of managers affect the level of corporate tax avoidance. The characteristics of management can be divided into external and internal factors. Educational background and workplace experience are external personal characteristics, whereas values, psychological orientation, and behavioral tendencies are internal personal characteristics. Internal characteristics can be shaped by various external experiences, and usually have more influence on the formulation of management decisions. Ishiguro and Yamada (2021) pointed out that overconfident management traits can motivate corporate tax avoidance. Graham et al. (2013) observed that the management attitude of managers profoundly affects the economic decisions made by the company. Tax avoidance has been regarded as a high-risk decision (Dhawan et al., 2020), covering credit risk, disclosure risk, and audit risk, which is manifested in a reduction in the transparency of financial reporting (Dyreng et al., 2010), the decline in a company’s credit rating, and an increase in audit attention.

Managers’ irrational expectations make them more optimistic about the risks of tax avoidance activities, causing them to overestimate the potential benefits and enhancing their willingness to guide tax avoidance decisions. Managers with irrational expectations are more confident about the future development of their enterprise and the impact of laws and regulations, and therefore they underestimate the risks of credit decline, reputational damage, and increased audit attention that are caused by tax avoidance activities. Accordingly, their irrational expectations will increase the company’s willingness to take risks. Kubick and Lockhart (2017) showed that the irrationality of overconfidence makes managers overestimate the likelihood that their decision-making will generate returns, which leads to a greater willingness to take risks. The result is that managers with irrational expectations increase not only the willingness of their enterprise to take tax avoidance risks but also the probability that it will make the decision to avoid tax. On the basis of these considerations, the following hypothesis is proposed:

*H1:* There is a significant positive correlation between management’s irrational expectations and the level of corporate tax avoidance.

The researches of Park and Kim (2020) have concluded that the personal characteristics and behavioral tendencies of management increase the level of risk-taking in enterprises, with corresponding economic consequences. According to upper echelons theory and behavioral economics theory, it is impossible for management to have a detailed understanding of all aspects of an enterprise. Management tends to focus on realizing its grand ambitions, paying too much attention to personal interests and neglecting macroeconomic factors; this narrow perspective leads to irrational bias in judgment and increases the risk-taking level of an enterprise. Empirical analysis has indicated that overconfident management traits promote the level of risk-taking of an enterprise, and that managers with overconfident traits are more optimistic about investment projects, which makes them adopt aggressive investment strategies under strong risk preference (Yu et al., 2013). Research on the subjective traits of managers, such as risk preference, has found that these traits can change with the intensity and direction of psychological bias, which further affects business decision-making and the risk-taking willingness of an enterprise (Hribar et al., 2017). As with the so-called emotional ripple effect, psychological deviation on the part of managers can generalize across the management environment to catalyze the behavioral costs in subsequent decision-making behavior. This phenomenon is prevalent when managers are in a relatively strong situation, regardless of whether their decision-making conforms to reality. Managers are often reluctant to change their minds and return to objective and rational thinking, so if they have over-optimistic, irrational expectations, they will lead their enterprises to make decisions that overestimate their risk-taking ability (Guo, 2009; Malmendier et al., 2011). The literature has also shown that managers with high risk preference hold irrational attitudes when running enterprises and are more inclined to increase corporate profits artificially (Abdel-Khalik, 2007; Grant et al., 2009). This risk preference has a significant impact on corporate performance smoothing and earnings management, whereas profit manipulation and earnings management affect tax fluctuations directly.

The level of risk-taking of an enterprise reflects its willingness to pay a price in order to obtain substantial profits through its business decisions (Lumpkin and Dess, 1996). On the one hand, enterprises with a higher level of risk-taking are more willing to make high-risk behavioral decisions. High-risk decision-making often has unpredictable economic consequences, and tax avoidance behavior is no exception, given the negative effects of compliance risk and reputation loss. On the other hand, the implementation of high-risk investment decisions demands a large amount of capital, which can lead to a shortage of funds and may affect the sustainable operation of an enterprise by increasing the cash holdings it requires. Tax avoidance can help to control the capital wealth in an enterprise in the short term by ensuring the stability of cash holdings and asset liquidity (Hanlon and Heitzman, 2010), and by alleviating any shortage of operating funds. On the basis of these considerations, the following hypothesis is proposed:

*H2:* Management’s irrational expectations increase the risk-taking level of an enterprise, thus affecting the enterprise’s tax avoidance decisions.

As an internal corporate governance mechanism, management shareholding is considered to effectively alleviate agency conflicts, that is, equity incentive for management can make the interests of management and the owners of enterprises more consistent, and reduce the moral hazard behavior of management (Tzioumis, 2008). The enterprise’s equity incentive to the management can produce a synergistic effect. The enterprise gives a certain proportion of equity to the management, which promotes the management to make decisions for the enterprise from the role of shareholder, and helps reduce the management’s behavior that damages the long-term interests of the enterprise by focusing only on the immediate interests (Jensen and Meckling, 1976). When the management equity incentive plan was implemented and management stockholding ratio would increase, which further reduced the management’s willing to undertake excessive risk (Lefebvre et al., 2010). The management holding corporate stocks will more carefully consider the impact of tax avoidance on the enterprise because tax avoidance is a risky management choice. Managers are more inclined to reduce the excessive risk from their own long-term interests. On the basis of these considerations, the following hypothesis is proposed:

*H3:* The managers’ stockholding plays a negative regulatory role in the impact of management’s irrational expectations on the level of tax avoidance.

**Figure 1:**
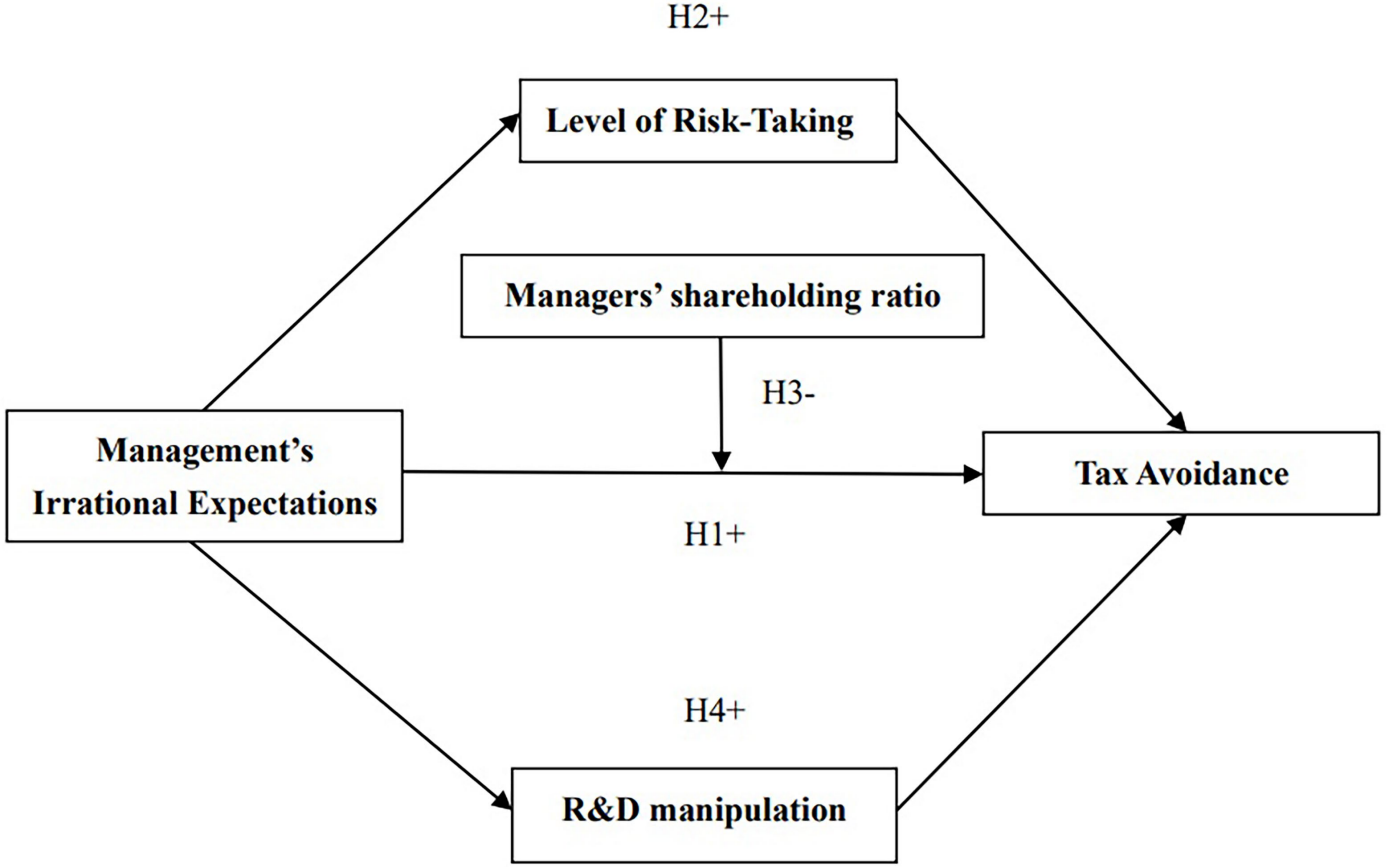
Conceptual model.

Some scholars have argued that management’s optimistic expression and overconfidence promotes self-interest and increases the possibility of earnings management and profit manipulation (Durana et al., 2022), thereby having an impact on tax avoidance. Nonetheless, few studies have explored the role of specific aspects of operating performance in such manipulation. Like other developing countries (Dobrzanski et al., 2021), given the strong incentives for R&D innovation in China’s current tax policy, some enterprises meet the criteria for high-tech enterprises by adjusting accounting subjects or by falsely inflating their R&D projects (Li et al., 2022). It is therefore likely that the characteristics of management may prompt enterprises to manipulate R&D in order to obtain policy advantages for the purposes of tax evasion (Figure 1). On the basis of these considerations, the following hypothesis is proposed:

*H4:* Management’s irrational expectations increase the R&D manipulation risk of an enterprise, thus affecting the enterprise’s tax avoidance decisions.

## Research design

### Choose the proper variables

This study takes the forward-looking text disclosed in the annual reports of listed companies as the information source. The forward-looking text is the information in the future outlook part of the MD&A section in an annual report. Irrational expectations of management are measured here in terms of excessive optimism of the disclosure text. Drawing on recent research (Feldman et al., 2010; Wang and Wang, 2018), a multiple regression analysis model was established based on the influencing factors of tone and dividing the net optimistic tone into two parts: normal tone and excessive tone. The residual represents the excessively optimistic part of management tone. Two kinds of specific influence factor models were used; one that considers the influence of the company’s future earnings and one that does not. The residuals of the two models are denoted by ABTONE (model 1) and ABTONE_FE (model 2). The specific models are as follows:

Model 1:


$$\begin{array}{l} \mathrm{Ton}e_{i,t}=\beta_{0}+\beta_{1}ROA_{i,t}+\beta_{2}\mathrm{LERE}T_{i,t}+\beta_{3}SIZE_{i,t}\\ +\beta_{4}BTM_{i,t}+\beta_{5}LEV_{i,t}+\beta_{6}STD\_RET_{i,t}\\ +\beta_{7}STD\_ROA_{i,t}+\beta_{8}AGE_{i,t}+\beta_{9}DIN_{i,t}\\ +\beta_{10}LOSS_{i,t}+\beta_{11}\mathrm{\Delta EAR}N_{i,t}+\varepsilon_{i,t} \end{array}$$

Model 2:


$$\begin{array}{l} \mathrm{Ton}e_{i,t}=\beta_{0}+\beta_{1}ROA_{i,t}+\beta_{2}\mathrm{LERE}T_{i,t}+\beta_{3}SIZE_{i,t}\\ +\beta_{4}BTM_{i,t}+\beta_{5}LEV_{i,t}+\beta_{6}STD_{R}ET_{i,t}\\ +\beta_{7}STD_{\_\_}ROA_{i,t}+\beta_{8}AGE_{i,t}+\beta_{9}DIN_{i,t}\\ +\beta_{10}LOSS_{i,t}+\beta_{11}\mathrm{\Delta EAR}N_{i,t}+\beta_{12}\mathrm{FEAR}N_{i,t}+\varepsilon_{i,t} \end{array}$$

ROA represents the return on total assets. LERET stands for the cumulative abnormal return of stocks calculated on a monthly basis. SIZE is the natural logarithm of the scale of total assets. BTM is book-to-market ratio. LEV is asset–liability ratio. STD_RET is the standard deviation of monthly stock returns. STD_ROA is the three-year standard deviation of the return on total assets. AGE represents the listing time of the company. DIN represents the number of industries involved in the main business of the enterprise, reflecting the degree of diversification of the company. LOSS is a dummy variable taking the value 1 for a loss in the current year, and 0 otherwise. ΔEARN represents the change in earnings for the year compared to the previous year. FEARN stands for future income (i.e., future return on total assets).

The dependent variable Net Tone is calculated as shown in model 3, with Posmda representing the number of optimistic words extracted from the forward-looking text and Negmda representing the number of pessimistic words.

Model 3:


$$\mathrm{Tone}=\frac{\mathrm{Posmda}-\mathrm{Negmda}}{\mathrm{Posmda}+\mathrm{Negmda}}$$

This study adopts an established method of calculating the level of an enterprise’s risk-taking (He et al., 2019), and establishes a modified model based on earnings volatility:

Model 4:


$$Adj\_Roa_{i,t}=\frac{EBIT_{i,t}}{\mathrm{ASSE}T_{i,t}}-\frac{1}{X}\overset{X}{\underset{k=1}{\sum }}\frac{EBIT_{i,t}}{\mathrm{ASSE}T_{i,t}}$$

$$\mathrm{Risk}1_{i,t}=\sqrt{\frac{1}{T-1}\overset{T}{\underset{t=1}{\sum }}{\left(Adj\_Roa_{i.t}-\frac{1}{T}\overset{T}{\underset{t=1}{\sum }}Adj\_Roa_{i,t}\right)}^{2}}|T=3$$

This model uses the degree of ROA volatility in the observed years to measure the level of risk-taking, such that the higher the volatility values, the higher the level of risk-taking. ROA denotes earnings before interest and taxes/total assets at year-end, as in the work of John et al. (2008). Adj_ROA was obtained by subtracting the annual industry average ROA from the ROA of each enterprise to reduce the impact of industry and cycle. The observation period was 3 years (*t* − 2 to *t*), and the standard deviation was calculated on a rolling basis. Finally, following the method of Faccio et al. (2011), the calculated results ×100 were used to generate risk indicators representing the level of enterprise risk-taking.

Drawing on previous research (Hanlon and Heitzman, 2010; Tian et al., 2019), the present study divides the measurement methods for tax avoidance indicators into the effective tax rate method and the tax difference method. Given the existence of many tax incentives and rebates for Chinese listed companies, the accuracy of using the effective tax rate method to measure tax avoidance is limited; this paper therefore uses the accounting-tax difference (BTD) method to measure corporate tax avoidance.

BTD = (pre-tax accounting profit−taxable income)/total assets at the end of the period

To ensure the accuracy of the BTD indicators, this study adopted the fixed residual method used by Desai and Dharmapala (2006) (Tian et al., 2019) to calculate the tax difference indicator (CBTD), excluding the impact of accruals in the current year and the impact of earnings management (Valaskova et al., 2021), as a measure of the level of tax aggressiveness of enterprises. The specific model is as follows:


$$BTD_{i,t}=\mathrm{\alpha TAC}C_{i,t}+\mu_{i}+\varepsilon_{i,t}$$

where TACC (accrued profit) = (net profit-net cash flow from operations)/total assets.$\mu_{i}$ is the mean of residuals of company *i* in the sample period. $\varepsilon_{i,t}$ is the residual of year *t* compared to the residual of the company’s deviation of mean value. CBTD = $\mu_{i}+\varepsilon_{i,t}$, the portion of the current year’s BTD that cannot be accounted for by TACC.

In line with previous research, when carrying out the principal regression hypothesis test this study controls for variables that affect corporate tax avoidance activities. Specific indicators include enterprise leverage (Lev; Kovacova et al., 2022), operating income growth ratio (Growth), tax collection and management intensity (TE), cash flow ratio (Cash), book-to-market ratio (BM), shareholding status of major shareholders (Top1), natural logarithm of asset size (LnSize), and Occupy and Year dummy variables for major shareholders. Table 1 gives the definitions of the variables.

**Table 1:** Definitions of primary variables.

Variable	Definition
CBTD	Tax avoidance level in the current year (excluding the impact of accrued profits)
ABTONE	Irrational intonation value (when future performance is not considered)
ABTONE_FE	Irrational intonation value (when considering future performance)
Risk	Corporate risk-taking level (calculated according to the method used by He et al., 2019)
Mshare	Managers’ shareholding ratio
RDIN	R&D manipulation (calculated according to the method used by Gunny, 2010)
LnSize	Natural logarithm of asset size
Lev	Total liabilities at end of period/total assets at end of period
TE	Regional tax collection intensity calculated according to the method proposed by Newlyn (2002)
Top1	Shareholding ratio of the largest shareholder
BM	Book value/total market value
Occupy	Other receivables divided by total assets
Cash	Net cash flow from operating activities/total assets
Growth	Operating income of current year/operating income of previous year −1

RDIN refers to the R&D manipulation of model measurement based on work by Gunny (2010) and Zhu et al. (2016).


$$\begin{array}{l} \frac{RD_{i,t}}{TA_{i,t-1}}=\alpha_{0}+\alpha_{1}\frac{1}{TA_{i,t-1}}+\alpha_{2}MV_{i,t}+\alpha_{3}TBQ_{i,t}\\ +\alpha_{4}\frac{\mathrm{IN}T_{i,t}}{TA_{i,t-1}}+\alpha_{5}\frac{RD_{i,t-1}}{TA_{i,t-1}}+\varepsilon_{i,t} \end{array}$$

$$\begin{array}{l} NMRD_{i,t}={\hat{\alpha }}_{0}+{\hat{\alpha }}_{1}\frac{1}{TA_{i,t-1}}+{\hat{\alpha }}_{2}MV_{i,t}+{\hat{\alpha }}_{3}TBQ_{i,t}\\ +\;{\hat{\alpha }}_{4}\frac{\mathrm{IN}T_{i,t}}{TA_{i,t-1}}+{\hat{\alpha }}_{5}\frac{RD_{i,t-1}}{TA_{i,t-1}} \end{array}$$

$$ABRD_{i,t}=\frac{RD_{i,t}}{TA_{i,t-1}}-\mathrm{NMRD}$$

TA represents total assets, RD represents R&D expenditure, MV represents the logarithm of the market value of the enterprise, and TBQ represents Tobin’s Q value. INT is operating profit, and NMRD is the normal R&D expenditure estimated from the model. Subtraction yields a residual ABRD, and the absolute value of ABRD is abnormal R&D manipulation. ABRD is then used to construct the R&D manipulation variable RDIN to test the relationship between analysts’ attention and R&D manipulation. Indep is the proportion of independent directors, RD is the R&D intensity (R&D expenditure/operating income), and INST is the proportion of institutional investors. Other variables are consistent with the definitions given in the section “Select the optimal data for the analysis.”

### Select the optimal data for the analysis

Since December 2005, the China Securities Regulatory Commission has officially updated its disclosure rules, requiring company annual reports to replace the Review and Outlook of Business Operations field with the MD&A field. To ensure a unified data standard, and avoid the impact of significant changes in tax policies caused by COVID-19 after 2020, in the present study, A-share listed companies affiliated to the Shanghai and Shenzhen Stock Exchanges from 2006 to 2020 were taken as the research sample for analysis. The number of industries involved and the relevant nominal tax rate data were taken from the Wind data terminal, and the contents of the MD&A fields were obtained from the China Research Data Service Platform. Other basic financial data were taken from the China Stock Market Accounting Research (CSMAR) database, and some data were integrated and calculated. Financial industry samples and company samples with Special Treatment (ST) marks were uniformly excluded to avoid the impact of extreme values and outliers. All continuous variables were treated at the upper and lower 1% levels.

### Construct the recursive model

In line with the mediating effect test methods used in previous empirical studies (Perera, 2013), this paper uses a recursive model to test whether management’s irrational expectations stimulate corporate tax avoidance activities and to measure the mediating effect of corporate risk-taking level:


$$\begin{array}{l} CBTD_{i,t}=\alpha_{0}+\alpha_{1}\mathrm{ABTon}e_{i,t}+\alpha_{2}Lev_{i,t}+\alpha_{3}\mathrm{Growt}h_{i,t}\\ +\alpha_{4}TE_{i,t}+\alpha_{5}Cash_{i,t}+\alpha_{6}BM_{i,t}+\alpha_{7}\mathrm{Top}1_{i,t}\\ +\alpha_{8}\mathrm{LnSiz}e_{i,t}+\alpha_{9}\mathrm{Occup}y_{i,t}+\sum \mathrm{year}+\varepsilon_{i,t} \end{array}$$

$$\begin{array}{l} Risk_{i,t}=\beta_{0}+\beta_{1}\mathrm{ABTon}e_{i,t}+\beta_{2}Lev_{i,t}+\beta_{3}ROE_{i,t}\\ +\beta_{4}\mathrm{Growt}h_{i,t}+\beta_{5}AGE_{i,t}+\beta_{6}AS_{i,t}+\beta_{7}Loss_{i,t}\\ +\beta_{8}\mathrm{LnSiz}e_{i,t}+\sum \mathrm{year}+\varepsilon_{i,t} \end{array}$$

$$\begin{array}{l} CBTD_{i,t}=\lambda_{0}+\lambda_{1}\mathrm{ABTon}e_{i,t}+\lambda_{2}Risk_{i,t}+\lambda_{3}Lev_{i,t}\\ +\lambda_{4}\mathrm{Growt}h_{i,t}+\lambda_{5}TE_{i,t}+\lambda_{6}Cash_{i,t}+\lambda_{7}BM_{i,t}\\ +\lambda_{8}\mathrm{Top}1_{i,t}+\lambda_{9}\mathrm{LnSiz}e_{i,t}+\lambda_{10}\mathrm{Occup}y_{i,t}\\ +\sum \mathrm{year}+\varepsilon_{i,t} \end{array}$$

Drawing on the hypothesis testing in the section “Multiple regression analysis,” this study therefore sets the Mshare variable to reflect the managers’ stockholding ratio of the listed companies, in order to test the moderating effect by adding a cross-multiplicative term.


$$\begin{array}{l} CBTD_{i,t}=\alpha_{0}+\alpha_{1}\mathrm{ABTon}e_{i,t}+\alpha_{2}\mathrm{Mshar}e_{i,t}+\alpha_{3}Lev_{i,t}\\ +\alpha_{4}\mathrm{Growt}h_{i,t}+\alpha_{5}TE_{i,t}+\alpha_{6}Cash_{i,t}+\alpha_{7}BM_{i,t}+\alpha_{8}\mathrm{Top}1_{i,t}\\ +\alpha_{9}\mathrm{LnSiz}e_{i,t}+\alpha_{10}\mathrm{Occup}y_{i,t}+\sum \mathrm{year}+\varepsilon_{i,t} \end{array}$$

$$\begin{array}{l} CBTD_{i,t}=\alpha_{0}+\alpha_{1}\mathrm{ABTon}e_{i,t}+\alpha_{2}\mathrm{Mshar}e_{i,t}+\alpha_{3}\mathrm{ABTone}\\ \times \mathrm{Mshar}e_{i,t}+\alpha_{4}Lev_{i,t}+\alpha_{5}\mathrm{Growt}h_{i,t}+\alpha_{6}TE_{i,t}+\alpha_{7}Cash_{i,t}\\ +\alpha_{8}BM_{i,t}+\alpha_{9}\mathrm{Top}1_{i,t}+\alpha_{10}\mathrm{LnSiz}e_{i,t}+\alpha_{11}\mathrm{Occup}y_{i,t}\\ +\sum \mathrm{year}+\varepsilon_{i,t} \end{array}$$

Drawing on previous research (Yuan et al., 2020), this study constructs the following model to test the impact of management’s irrational expectations on R&D manipulation:


$$\begin{array}{l} RDIN_{i,t}=\alpha_{0}+\alpha_{1}\mathrm{ABTon}e_{i,t}+\alpha_{2}Lev_{i,t}+\alpha_{3}\mathrm{Growt}h_{i,t}\\ +\alpha_{4}TE_{i,t}+\alpha_{5}Cash_{i,t}+\alpha_{6}BM_{i,t}+\alpha_{7}\mathrm{Top}1_{i,t}+\alpha_{8}\mathrm{LnSiz}e_{i,t}\\ +\alpha_{9}\mathrm{Occup}y_{i,t}+\sum \mathrm{year}+\varepsilon_{i,t} \end{array}$$

$$\begin{array}{l} CBTD_{i,t}=\lambda_{0}+\lambda_{1}\mathrm{ABTon}e_{i,t}+\lambda_{2}RDIN_{i,t}+\lambda_{3}Lev_{i,t}\\ +\lambda_{4}\mathrm{Growt}h_{i,t}+\lambda_{5}TE_{i,t}+\lambda_{6}Cash_{i,t}+\lambda_{7}BM_{i,t}+\lambda_{8}\mathrm{Top}1_{i,t}\\ +\lambda_{9}\mathrm{LnSiz}e_{i,t}+\lambda_{10}\mathrm{Occup}y_{i,t}+\sum \mathrm{year}+\varepsilon_{i,t} \end{array}$$

## Empirical results analysis

### Descriptive statistics

Table 2 gives the descriptive statistics for all the variables in the model. The mean value of tax avoidance level (CBTD) of enterprises excluding accrued profits in the current year is−0.0088, the standard deviation is 0.12, and the median approximation is 0.00, which indicates both that tax avoidance behavior is prevalent and that there are differences in tax avoidance activities among the enterprises in the sample. The average value of irrational expectations (ABTONE), measured in terms of the tone of management in forward-looking texts, is −0.0229, with a median of −0.02 and a maximum of 0.37, which indicates that a certain number of enterprises have irrational and over-optimistic expectations. The average value for level of risk-taking (Risk) is 0.0229, with a maximum of 0.27 and a median of only 0.01, which indicates that the differences among enterprises in level of risk-taking are high and distributed toward the right. In addition, the average and median of the largest shareholder’s shareholding ratio (Top1) are 34.0927 and 31.98, respectively, indicating that there are a large number of dominant shares in the sample companies. Other control variables are within the normal reference range.

**Table 2:** Descriptive statistics.

VarName	Obs	Mean	SD	Min	Median	Max
CBTD	15,335	−0.0088	0.120	−0.54	0.00	0.24
ABTONE	15,335	−0.0229	0.118	−0.35	−0.02	0.37
Risk	15,335	0.0229	0.027	0.00	0.01	0.27
RDIN	15,335	0.0055	0.007	0.00	0.00	0.07
Mshare	15,335	0.0705	0.143	0.00	0.00	0.65
LnSize	15,335	22.5208	1.292	19.52	22.35	26.40
Lev	15,335	0.4499	0.198	0.06	0.45	0.99
TE	15,335	1.0030	0.208	0.55	0.98	1.58
Top1	15,335	34.0927	14.639	7.93	31.98	76.95
BM	15,335	1.2137	1.311	0.05	0.78	9.95
Occupy	15,335	0.0163	0.024	0.00	0.01	0.20
Cash	15,335	0.0512	0.068	−0.20	0.05	0.26
Growth	15,335	0.1885	0.473	−0.73	0.10	4.71

### Multiple regression analysis

The three-stage method of regression was used to determine how the irrational expectations of management affected the tax avoidance behavior of enterprises (see Table 3 for the results). Model (1) verifies that management’s irrational expectations can reduce corporate tax avoidance behavior. The coefficient of ABTONE is 0.0430, significantly positive at the level of 1%, which indicates that management’s irrational expectations can significantly increase the degree of aggressive corporate tax avoidance. H1 is therefore supported. In model (2), ABTONE and Risk are significantly positive at the level of 1%, which indicates that ABTONE can increase risk-taking. In model (3), where the level of enterprise risk-taking (Risk) is added to model (1), the coefficient of management irrational expectation (ABTONE) and the level of enterprise risk-taking (Risk) are also significantly positive at the 1% level. The coefficient of management’s irrational expectation decreases from 0.0452 in model (1) to 0.0430 in model (3), which indicates that the level of corporate risk-taking plays a strong mediating role in the impact of management’s irrational expectation on corporate tax avoidance behavior.

**Table 3:** Mediating effect of corporate risk-taking level.

	(1)	(2)	(3)
	CBTD	Risk	CBTD
ABTONE	0.0452^***^	0.0117^***^	0.0430^***^
	(6.2673)	(6.5417)	(5.9759)
Lev	−0.0894^***^	0.0308^***^	−0.0904^***^
	(−15.7503)	(22.9188)	(−15.9157)
Growth	0.0117^***^	−0.0009^*^	0.0110^***^
	(6.5704)	(−1.9015)	(6.1059)
TE	−0.0038	0.0031^***^	−0.0036
	(−0.8174)	(2.6553)	(−0.7814)
Cash	0.0048	−0.0058^***^	0.0043
	(0.5770)	(−2.7010)	(0.5134)
BM	−0.0143^***^	−0.0043^***^	−0.0137^***^
	(−14.1589)	(−16.4828)	(−13.5736)
Top1	0.0000	−0.0001^***^	0.0000
	(0.5486)	(−8.6284)	(0.7194)
LnSize	0.0151^***^	−0.0062^***^	0.0154^***^
	(16.2593)	(−26.3283)	(16.5009)
Occupy	−0.3191^***^	0.1486^***^	−0.3294^***^
	(−9.9310)	(20.8821)	(−10.2334)
Risk			0.1564^***^
			(4.7268)
_cons	−0.1458^***^	0.2499^***^	−0.1803^***^
	(−2.9399)	(24.4264)	(−3.6127)
Sobel Z			−5.631^***^
*N*	15,335	15,335	15,335
adj. *R*^2^	0.052	0.116	0.053

*t* statistics in parentheses.

^*^p < 0.1, ^**^p < 0.05, and ^***^p < 0.01.

The results of the Sobel test show that the Z value is also significant at the 1% level, and thus H2 is supported. These empirical results permit the conclusion that the irrational expectations of management enhance the risk preference of business decision-making through their own decision-making influence, enhance the risk-taking level of enterprises indirectly, and stimulate the tax avoidance behavior of enterprises. With the aim of obtaining greater liquidity, companies seek to take advantage of the complexity of transactions and of loopholes in laws and regulations to manipulate their surplus and profits, increase their risk-taking, and thus increase their ability to avoid taxes by relying on asymmetric information.

### Moderating effect analysis

The present study has thus far focused on the impact of management’s irrational expectations on levels of corporate tax avoidance. Some studies, however, have suggested that the managers’ stockholding ratio in enterprises has a significant impact on the role of management’s personal behavioral tendencies (Lefebvre et al., 2010). The results are shown in Table 4. The ABTONE × Mshare coefficient of management’s irrational expectations and the nature of corporate property rights is significantly negative (β = −0.0900, *p* < 0.10), which indicates that the degree of management’s irrational expectations has a more significant impact on tax avoidance behavior when manager holding less stock of enterprise. This conclusion runs consistent with H3. With reference to previous research, the following reasons for this finding can be adduced. The problem of corporate governance results from the separation of ownership in the modem company, so that the interests of shareholders and managers conflict. Therefore, designing an effective incentive contract to get managers’ behavior goals in accordance with shareholders’ interests could effectively alleviate this conflict. Jensen and Meckling (1976) pointed out that the relationship between management shareholding and incentive intensity is linear, and management shareholding could promote the generation of interest convergence effect. From a psychological perspective, awarding a certain proportion of shares to the management will establish a benefit oriented psychological contract between the management and the company’s shareholders. This psychological contract is invisible, dynamic and subjective, it is impossible to describe and limit in the form of express terms. On the one hand, the grant of equity represents a kind of material power; on the other hand, it also represents a kind of decision-making power. This power gives shareholders and management more trust invisibly, so the management may be more cautious in making decisions, indirectly controlling the negative impact of their irrational expectations.

**Table 4:** Moderating effect of the managers’ stockholding ratio.

	(2)	(3)
	CBTD	CBTD
ABTONE	0.0431^***^	0.0347^***^
	(5.9813)	(4.5501)
Lev	−0.0888^***^	−0.0897^***^
	(−15.5785)	(−16.1279)
Growth	0.0116^***^	0.0111^***^
	(6.4929)	(6.2558)
TE	−0.0037	−0.0192^***^
	(−0.7956)	(−4.6287)
Cash	0.0052	0.0041
	(0.6213)	(0.4862)
BM	−0.0142^***^	−0.0120^***^
	(−14.1146)	(−12.4826)
Top1	0.0000	0.0001
	(0.6219)	(1.3378)
LnSize	0.0152^***^	0.0128^***^
	(16.2628)	(14.7996)
Occupy	−0.3182^***^	−0.2868^***^
	(−9.8997)	(−8.9572)
Mshare	0.0057	−0.0066
	(0.8986)	(−1.0330)
ABTONE × Mshare		−0.0900^*^
		(−1.7231)
_cons	−0.2841^***^	−0.2199^***^
	(−14.1597)	(−11.6705)
*N*	15,335	15,335
adj. *R*^2^	0.052	0.050

*t* statistics in parentheses.

^*^p < 0.1, ^**^p < 0.05, and ^***^p < 0.01.

### Expansibility test results

Table 5 shows the results of the regression regarding impact on R&D Manipulation. There is a significant positive correlation between management’s irrational expectations and R&D manipulation, and management’s irrational expectations significantly increase a company’s level of R&D manipulation. Consistent with the literature, there is a significant positive correlation between R&D manipulation and the level of corporate tax avoidance. These results therefore confirm the conclusion of this study that management’s irrational expectations stimulate corporate tax avoidance.

**Table 5:** Impact of managerial irrational expectations on R&D manipulation.

	(1)	(2)
	RDIN	CBTD
ABTONE	0.0008^*^	0.0643^***^
	(1.8269)	(7.1173)
Lev	0.0006^*^	−0.0775^***^
	(1.7907)	(−11.0570)
Growth	0.0032^***^	0.0147^***^
	(24.8835)	(5.7440)
TE	0.0006^**^	−0.0174^***^
	(2.3689)	(−3.1248)
Cash	0.0052^***^	0.0144
	(9.6028)	(1.3986)
BM	−0.0005^***^	−0.0131^***^
	(−8.2975)	(−10.8450)
Top1	0.0000^**^	−0.0000
	(2.0982)	(−0.6067)
LnSize	−0.0002^***^	0.0127^***^
	(−3.8584)	(11.4260)
Occupy	−0.0003	−0.3460^***^
	(−0.1494)	(−7.4279)
RDIN		0.6478^***^
		(4.2035)
_cons	0.0085^***^	−0.2250^***^
	(7.0965)	(−9.5322)
*N*	15,335	15,335
adj. *R*^2^	0.063	0.051

*t* statistics in parentheses.

^*^p < 0.1, ^**^p < 0.05, and ^***^p < 0.01.

### Robustness tests

This study used two methods of robustness testing. Because the annual report disclosure had in most cases entered the next business year, it was not accurate enough to measure the management’s irrational expectations without taking into account the impact of future annual earnings. Therefore, this study included management’s irrational expectations (ABTONE_FE) after controlling for the company’s future earnings as an independent variable to substitute into the original regression model. Similarly, because the tax difference may not fully reflect the degree of corporate tax avoidance, this study used the effective tax rate, replacing the difference between the nominal tax rate and the actual tax rate (Ratediff) as the dependent variable for purposes of robustness testing. As shown in Tables 6, 7, the results are consistent with the results of the main testing, which support the research conclusions of this study.

**Table 6:** Mediating effect after replacement of explanatory variables.

	(1)	(2)	(3)
	CBTD	Risk	CBTD
ABTONE_FE	0.0342^***^	0.0076^***^	0.0360^***^
	(4.4936)	(4.4553)	(4.7157)
Lev	−0.0918^***^	0.0238^***^	−0.0922^***^
	(−15.0562)	(18.1914)	(−15.1143)
Growth	0.0108^***^	0.0003	0.0104^***^
	(5.8273)	(0.7432)	(5.5309)
TE	−0.0041	0.0026^**^	−0.0039
	(−0.8658)	(2.4033)	(−0.8204)
Cash	0.0032	−0.0053^***^	0.0030
	(0.3631)	(−2.6203)	(0.3407)
BM	−0.0154^***^	−0.0043^***^	−0.0150^***^
	(−13.0958)	(−15.8475)	(−12.6808)
Top1	0.0000	−0.0001^***^	0.0000
	(0.6027)	(−5.6430)	(0.6568)
LnSize	0.0162^***^	−0.0051^***^	0.0163^***^
	(16.1585)	(−22.1453)	(16.1934)
Occupy	−0.3255^***^	0.1205^***^	−0.3312^***^
	(−9.6129)	(17.6343)	(−9.7663)
Risk			0.1108^***^
			(2.7845)
_cons	−0.3050^***^	0.1328^***^	−0.3100^***^
	(−14.2891)	(27.3554)	(−14.4125)
*N*	15,335	15,335	15,335
adj. *R*^2^	0.051	0.099	0.052

*t* statistics in parentheses.

^*^p < 0.1, ^**^p < 0.05, and ^***^p < 0.01.

**Table 7:** Mediating effect test after replacement of the explained variable.

	(1)	(2)	(3)
	Ratediff	Risk	Ratediff
ABTONE	0.0430^***^	0.0117^***^	0.0452^***^
	(5.9760)	(6.5417)	(6.2676)
Lev	−0.0894^***^	0.0308^***^	−0.0904^***^
	(−15.7507)	(22.9188)	(−15.9162)
Growth	0.0117^***^	−0.0009^*^	0.0110^***^
	(6.5711)	(−1.9015)	(6.1065)
TE	−0.0038	0.0031^***^	−0.0036
	(−0.8173)	(2.6553)	(−0.7812)
Cash	0.0048	−0.0058^***^	0.0043
	(0.5762)	(−2.7010)	(0.5126)
BM	−0.0143^***^	−0.0043^***^	−0.0137^***^
	(−14.1591)	(−16.4828)	(−13.5737)
Top1	0.0000	−0.0001^***^	0.0000
	(0.5483)	(−8.6284)	(0.7192)
LnSize	0.0151^***^	−0.0062^***^	0.0154^***^
	(16.2591)	(−26.3283)	(16.5008)
Occupy	−0.3191^***^	0.1486^***^	−0.3294^***^
	(−9.9300)	(20.8821)	(−10.2324)
Risk			0.1565^***^
			(4.7275)
_cons	−0.2812^***^	0.1570^***^	−0.2916^***^
	(−14.2057)	(31.4176)	(−14.6051)
*N*	15,335	15,335	15,335
adj. *R*^2^	0.052	0.116	0.053

*t* statistics in parentheses.

^*^p < 0.1, ^**^p < 0.05, and ^***^p < 0.01.

## Discussion

The irrational expectations of managers can lead to deviations in investment decisions, higher levels of risk-taking by enterprises, and increased transaction complexity and profit manipulation, thereby affecting the level of tax avoidance of enterprises. Previous research has largely ignored the impact of the irrational expectations of managers on levels of risk-taking and tax avoidance. The present study is therefore of significance in addressing this research gap on the mechanisms of corporate tax avoidance. Actual cases have also confirmed this conclusion. According to Sina, there are tens or even hundreds of listed companies that are subject to tax penalties owing to the personal decisions of the management. For instance, Shenzhen-listed Ningxia Zhong yin Cashmere Company responded to investors in 2016 after receiving a tax concern letter from the Shenzhen Stock Exchange: Ma Shengguo, the chairman of the board in 2016, acknowledged in the disclosure that he previously used his position as the chairman and general manager of your company, as well as the company’s export tax refund qualification, to falsely report exports with others without following their company’s internal review and other decision-making procedures. The tax refund obtained is about 120 million RMB. Through the disclosure of the annual report of the previous year, we extracted the number of emotional words in the annual report, and we came up with a total of 164 positive words. Despite the company experiencing repeated years of declining net profits, the MD&A disclosure of the management still showed excessive positive expressions such as “increase,” “expansion,” “turn a loss into profit,” etc. Calculations revealed that the calculated emotional intonation value of the year was −0.0151, and the intonation value changed significantly higher in comparison to the previous year’s − 0.0255. In the following years, Shenzhen Stock Exchange repeatedly sent letters of concern to the company. The problems cited included incomplete information disclosure in the annual report and the content of the disclosure was inconsistent with the facts. Further review of news media reports revealed that Mr. Ma, the former chairman of the board, was also subject to administrative penalties multiple times. He was frequently accused of taking part in high-risk foreign trade trading activities through subsidiaries and was included in the list of dishonest people several times. In 2017–2019, the company’s shares continued to be marked with ST (Special Treatment for delisting risk), meaning that its annual profits were negative. This indicates that the risk preference, as well as the personal characteristics of managers, can greatly impact the level of enterprise risk bearing, and managers can put the entire enterprise into a difficult situation by making high-risk decisions. On the other hand, investors can perceive this risk in the managers’ irrational emotional expression in the annual report. With the consent of the controlling shareholder Hengtian Jinshi and the general meeting of shareholders, the company’s board of directors along with senior management was replaced in 2020. In the annual report, the MD&A’s emotional intonation value dropped to −0.0108, while the degree of rationality increased dramatically. The degree of tax avoidance changed from 0.02 to −0.01. The company’s profits in 2021 became positive. The ST logo was removed, and the stock price rose steadily, further demonstrating the significance of the management’s rational behavior in the company’s decision-making and growth.

Because the irrational expectations of management are not easy to observe, most studies have been limited to theoretical elaboration and single-case analysis, with a biased selection of indicators that cannot adequately measure the emotional color of the irrational expectations, despite recent advances in natural language processing technology. However, given the text information disclosures made by listed companies under the requirements of the Securities Regulatory Commission, the irrational expectations of management can now be understood by capital market participants through voice analysis of the forward-looking text information disclosed by enterprises. The present study uses this developing research context to provide new evidence for the economic consequences of management’s irrational expectations. The effectiveness of the disclosures contained in the management’s discussion and analysis (MD&A) sections of the annual reports of listed companies in China has received the attention of many academic studies. The present study makes use of this disclosure to clarify the inherent meaning of the regulation and to explore the potential economic consequences, both foreseen and unforeseen. The results will help investors and regulators to understand and use text disclosure information more effectively in their decision-making. This also proves that it is of great significance for the China Securities Regulatory Commission to make strict disclosure requirements for listed companies. By analyzing the management disclosure, the supervision of management and enterprise operation can be better implemented, so as to protect the interests of investors and safeguard social fairness, for example, taxation fairness.

## Concluding remarks

Using a sample of A-share listed companies on the Shanghai and Shenzhen Stock Exchanges from 2006 to 2020, this study excluded accruals from accounting and tax differences to measure the degree of corporate tax avoidance. It examined the relationship between management’s irrational expectations and corporate tax avoidance levels, taking into account the level of corporate risk-taking and the nature of corporate property rights. The results show (1) that, in general, the irrational expectations of management significantly stimulate corporate tax avoidance; (2) that the irrational expectations of management increase the level of risk-taking of enterprises in relation to tax avoidance; that is, the level of risk-taking of enterprises has an intermediary effect between the irrational expectations of management and the level of tax avoidance of the enterprise; and (3) that the managers’ stockholding ratio play a moderating role in the impact of the irrational expectations of management on corporate tax avoidance behavior, in that the managers’ stockholding mitigates this effect. Moreover, due to the substantial tax incentives that R&D expenditure brings to enterprises, R&D manipulation for tax avoidance purposes is widespread. Through empirical testing, this study establishes that management’s irrational expectations have a significant impact on the level of R&D manipulation carried out by enterprises.

This study makes two main contributions to the literature. First, previous studies on the characteristics of management, limited by the availability of data, focused on the background factors of management, risk preferences, and overconfidence, rather than exploring how the characteristics of management affect corporate decision-making in relation to judgments of corporate future prospects. Because the common goal of enterprise decision-making is to help an enterprise to operate and thrive in the future, the management’s outlook and expectations play an important role in an enterprise’s development. This study analyzes the governance effect of irrational expectations based on management’s forward-looking statements, thereby providing further evidence for the objective observation of the economic consequences of management’s irrational expectations. Second, previous studies also focused on the capital market effects of management characteristics, such as financial crisis, enterprise value, and stock price crash, ignoring the governance risks associated with management characteristics. The findings of the present research go beyond this, supporting the conclusion that the irrational expectations of management significantly stimulate corporate risk taking and tax avoidance behavior. The findings also add to the evidence that management characteristics have direct economic consequences, notably in the form of manipulation of business data and concealment of the true business status of an enterprise.

Based on the research conclusions, we put forward some substantive suggestions. For enterprises, the internal governance environment should be further improved. On the one hand, enterprises should actively adopt material incentives such as equity incentives to establish a psychological contract between the management and shareholders, which enhance the mutual trust between the managers and the actual controllers of the enterprise, so that the managers can consider the long-term interests of the enterprise to a certain extent and refuse to make aggressive tax avoidance decisions. On the other hand, it is necessary to establish a risk early warning mechanism for management’s abnormal decisions and pay attention to adjusting the management’s composition structure, and stabilize the advantages of human resources. It is significant to increase emotional assessment items in the daily supervision of managers, so that managers can make decisions under a relatively rational condition. This kind of emotional assessment should especially include the risk appetite test of the management in order to avoid changing the overall risk tolerance level of the enterprise due to the “emotional ripple” effect. For regulators (policy makers), the enterprise information disclosure system should be further improved. The aforementioned conclusion indicates that enterprises may carry out radical tax avoidance through R&D manipulation. Therefore, the tax authorities should undertake a more rigorous evaluation while conducting tax credits and refunds related to R&D, and necessitate enterprises to disclose detailed R&D records to verify that R&D expenditure actually occurs and is conducive to enterprises’ future economic benefits. As far as the government is concerned, it should keep taking initiatives to advocate reasonable tax avoidance, encourage sustainable development of enterprises, and appropriately reward and publicize enterprises with high tax rates (Hamzah et al., 2021). Regularly convene key management personnel of the enterprise to discuss and foster a culture where everyone is aware of the importance of paying taxes on time and abiding by the provisions of the tax law. Moreover, it is crucial for investors, particularly institutional ones, to carefully read the annual disclosure report of listed firms. In addition to paying attention to the disclosed financial indicators, they should also take into account non-financial information, such as the words and expressions of managers, assess whether the company’s expected operating conditions expressed by them differ significantly from the digital indicators presented, and accurately judge the real investment value of the company. Finally, from managers perspective, although tax Planning is a sort of widely recognized legitimate Tax Saving behavior, it needs to be faced with a professional and rational attitude, aggressive tax avoidance in an irrational state would eventually lead to legal punishment and jeopardize managers’ own interests.

## Limitations and future work

Our findings provide a solid basis for future research on the topic of management irrational expectation on negative corporate decisions such as tax avoidance. But we must also point out that this study includes limitations that should be addressed in future research. One of the main shortcomings is that its measurement of managers’ irrationality is limited to their emotional words of text disclosure in official documents, which limits the external validity of the research results. Future research can test the driving effect of managers’ irrational expectations on tax avoidance behavior together with other variables, which will make the results more reliable. For example, the difference between the management’s prediction of future profits and the actual profits. Secondly, this research only points out two intermediary variables, namely, the level of enterprise risk bearing and R&D manipulation. Many other factors, such as differences in enterprise strategic choices, governance structures, and decision-making system, may play an important role in managers’ irrational and aggressive tax avoidance, which are expected to be promoted in subsequent researches. Finally, as tax avoidance issues are more common around the world, the study is expected to be replicated in different cultural backgrounds and different samples outside China to improve universality.

## Data availability statement

The raw data supporting the conclusions of this article will be made available by the authors, without undue reservation.

## Author contributions

LL drafted this manuscript. QW revised this draft. All authors contributed to the article and approved the submitted version.

## Funding

This work was supported by The Post-Funded Projects of The National Social Science Fund of China (Grant No. 21FJYB046) and The Humanities and Social Sciences Fund of the Ministry of Education (Grant No. 21YJA790076).

## Conflict of interest

The authors declare that the research was conducted in the absence of any commercial or financial relationships that could be construed as a potential conflict of interest.

## Publisher’s note

All claims expressed in this article are solely those of the authors and do not necessarily represent those of their affiliated organizations, or those of the publisher, the editors and the reviewers. Any product that may be evaluated in this article, or claim that may be made by its manufacturer, is not guaranteed or endorsed by the publisher.
